# Dual Role of the Alternative Reading Frame ARF Protein in Cancer

**DOI:** 10.3390/biom9030087

**Published:** 2019-03-04

**Authors:** Rosa Fontana, Michela Ranieri, Girolama La Mantia, Maria Vivo

**Affiliations:** 1 Department of Pharmacology, Moores Cancer Center, University of California, San Diego, La Jolla, CA 92093, USA; rofontana@ucsd.edu; 2 Division of Hematology and Medical Oncology, Laura and Isaac Perlmutter Cancer Center, NYU Langone Medical Center, New York, NY 10016, USA; Michela.Ranieri@nyulangone.org; 3 Department of Biology, University of Naples Federico II, 80126 Naples, Italy; lamantia@unina.it

**Keywords:** tumor suppression, autophagy, anoikis, *CDKN2a*/*ARF* locus, chemoresistance, FAK sumoylation, actin cytoskeleton

## Abstract

The CDKN2a/ARF locus expresses two partially overlapping transcripts that encode two distinct proteins, namely p14ARF (p19Arf in mouse) and p16INK4a, which present no sequence identity. Initial data obtained in mice showed that both proteins are potent tumor suppressors. In line with a tumor-suppressive role, ARF-deficient mice develop lymphomas, sarcomas, and adenocarcinomas, with a median survival rate of one year of age. In humans, the importance of ARF inactivation in cancer is less clear whereas a more obvious role has been documented for p16INK4a. Indeed, many alterations in human tumors result in the elimination of the entire locus, while the majority of point mutations affect p16INK4a. Nevertheless, specific mutations of p14ARF have been described in different types of human cancers such as colorectal and gastric carcinomas, melanoma and glioblastoma. The activity of the tumor suppressor ARF has been shown to rely on both p53-dependent and independent functions. However, novel data collected in the last years has challenged the traditional and established role of this protein as a tumor suppressor. In particular, tumors retaining ARF expression evolve to metastatic and invasive phenotypes and in humans are associated with a poor prognosis. In this review, the recent evidence and the molecular mechanisms of a novel role played by ARF will be presented and discussed, both in pathological and physiological contexts.

## 1. Introduction

**Figure 1:**
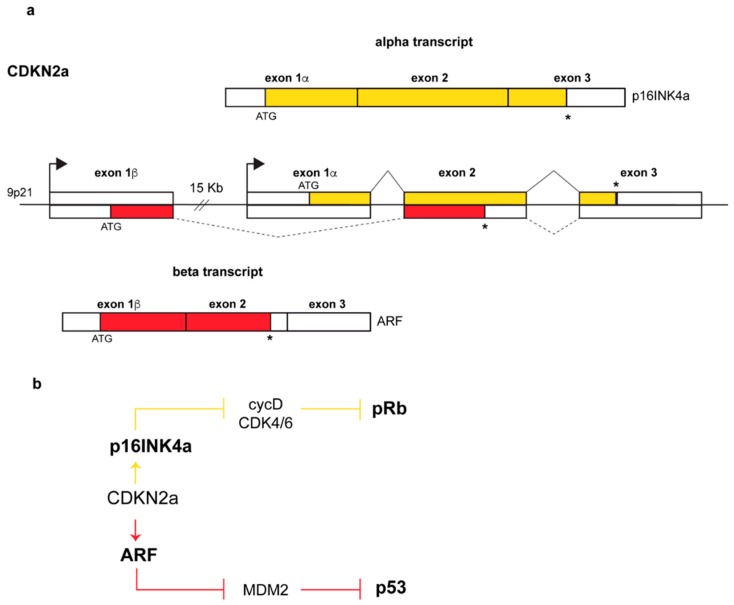
Genomic structure of the CDKN2a locus and produced transcripts. (**a**) Arrows above each exon 1 indicate promoters, continuous and dashed lines above and below the genomic structure indicate *p16* and *ARF* splicing patterns respectively. Transcription of exon 1β, and its splicing to exons 2 and 3 results in the α-transcript, encoding p16INK4a, whereas transcription starting upstream of exon 1β produces the β-transcript in which the exon1β, and the common exons 2 and 3 encode ARF (p14ARF in human, p19Arf in mouse). In yellow and in red are indicated the open reading frames (ORFs) of p16 and ARF respectively, with exon 2 displaying two overlapped ORFs. White boxes represent untranslated regions at the 3’ and 5’ ends while asterisks (*) indicate stop codons (**b**) Pathways regulated by the two proteins: while p14ARF inhibits Mdm2 (Mouse Double Minute-2) functions with consequential p53 stabilization [4,5], p16INK4a inhibits the cyclinD-CDK4/6 complex thus maintaining the retinoblastoma protein pRb in its growth-suppressive mode [4].

The ARF (alternative reading frame) protein is encoded by the Alternative Reading Frame of the *CDKN2a* locus, one of the most frequently mutated sites in human cancers after the p53 locus [1,2,3]. The locus, located on human chromosome 9p21, encodes two completely unrelated proteins, p16INK4a and p14ARF, both of which are potent tumor suppressors. The mechanism by which the two proteins are produced is quite unusual. Each gene is endowed with its own promoter that guides the transcription of an α- or β-transcript. Each transcript has a specific 5’ exon, E1α or E1β for INK4a and ARF respectively, spliced to a common exon 2 (Figure 1a) in which two overlapped ORFs (Open Reading Frame) are translated into two proteins sharing no amino acid sequence identity at all.

The alpha transcript encodes the p16INK4a protein, a member of the INK4 family of inhibitors of the cyclin-dependent kinases 4 and 6 (Inhibitor of CDK4). In response to specific signals, they block the assembly and/or inhibit the kinase activity of the cyclin D-CDK4/6 complex required for G1 to S cell cycle progression [6,7]. In this way, the retinoblastoma protein pRB is maintained in an active hypo-phosphorylated state and sequesters the transcription factors of the E2F family causing G1-phase cell cycle arrest [7,8] (Figure 1b). The ARF protein instead inhibits the functions of the MDM2 oncoprotein (Mouse Double Minute 2, HDM2 in human) thus inducing p53 stabilization and the activation of p53-dependent pathways (Figure 1b).

In humans, the β transcript results in a polypeptide of 132 amino acids (14 kDa) named p14ARF while, in mice, the transcript is translated into a 169 amino acid polypeptide named p19ARF (19kDa). Human and mouse proteins share only 50% of identity. Interestingly, the exon 1β-encoded N-terminal region, that is necessary and sufficient to fulfil almost all of the known ARF tumor suppressor functions, is only modestly conserved between species, whereas the exon 2-encoded C-terminal region shows a stronger degree of identity between human and mouse (57% of identity) [5]. By comparison, mouse and human INK4a are more conserved, sharing the 65% of identity overall [9]. ARF proteins are highly basic (> 20% arginine content) and hydrophobic molecules. The basic nature of ARF renders this protein highly insoluble and this is likely the reason for which neither NMR (nuclear magnetic resonance) nor crystal structure has been determined, despite its small size. ARF probably needs to form complexes with other molecules to assume specific spatial conformation and to neutralize its charge at physiological pH, thus explaining the incredible number of ARF binding partners [10].

ARF is a potent tumor suppressor, regulating cell cycle arrest and/or apoptosis by both p53-dependent and independent pathways [11]. Interestingly, it represents a link between the pRb and the p53 pathway, the most important tumor suppressor pathways within the cell. It has been demonstrated the ARF triggers a p53-dependent checkpoint when the pRb pathway is compromised. In particular, when inactivated, pRb causes the release of E2F transcription factors, which in turn induce the increase of ARF transcription [5,12].

The classical ARF tumor suppressor function has been challenged by the recent observations suggesting that this protein is overexpressed and plays pro-oncogenic functions in specific types of human cancers. In this review, ARF dependent tumor suppressor mechanisms will be described together with the new evidence outlining its pro-survival functions.

## 2. ARF Inactivation in Human and Murine Cancer

A contribution of ARF to tumor formation has been documented using genetic analysis of tumors, molecular and cell biology approaches, and animal models [13,14]. In respect to p16, ARF seems to assume a more prominent role as a tumor suppressor in mice rather than in humans, where the mutation frequency of this gene is low. *Arf*-null mice usually die after one year since they develop tumors, mostly sarcomas (43%), lymphoid malignancies (29%), carcinomas (17%), and tumors of the nervous system (11%). Furthermore, tumors arising in the heterozygous mice undergo further deletion of the remaining allele [15].

In most cases of human cancers, both ARF and p16 are inactivated, making it difficult to determine their individual roles in tumor suppression [16,17,18,19,20]. Alterations of the *CDKN2a* locus were found in roughly 30% of human tumors such as glioblastoma, melanoma and pancreatic adenocarcinoma [8,21]. However, there are specific examples in which only *ARF* appears to be affected in human cancer. Promoter methylation in *ARF* gene has been reported to occur independently of the *INK4a* promoter methylation, thus suggesting that this is a specific alteration of ARF expression. A CpG island within the promoter region of *p14ARF* has been found to be hyper-methylated [21] in a wide spectrum of human cancers such as colorectal [22], gastric [23] and prostate carcinomas [24]. Deletions and point mutations of the exon 1β has been reported in familial melanoma syndromes and in glioblastoma [11,17,25,26,27,28]. In breast cancer, several insults such as homozygous deletion, loss of heterozygosity and promoter hyper-methylation, have all been described [29]. Interestingly, in the majority of non-small cell lung cancer (NSCLC), p14ARF protein is expressed at low levels, albeit no genetic mutations or known transcriptional alterations have been reported so far [30].

## 3. ARF is Involved in the p53 Pathway

**Figure 2:**
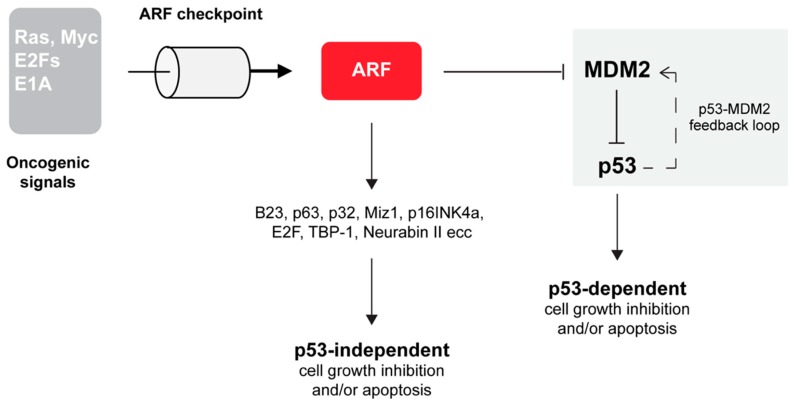
ARF involvement in tumor suppression relies on p53-dependent and independent pathways. Upon over-expression of oncogenes (such as E2F, Ras, E1A and Myc), an increase of ARF intracellular levels are promptly observed (ARF checkpoint) in the cell. By inhibiting MDM2 functions, ARF interferes with the p53/MDM2 circuit (highlighted in grey), leading to p53 stabilization followed by cell cycle arrest and/or apoptosis [10,32,33,34,35,39]. ARF is able to block cell growth also in a p53-independent manner, through the functional interaction with several molecular players as indicated.

One of the most well-defined ARF function is the suppression of aberrant cell growth in response to oncogene activation [12,31,32,33,34]. In particular, in this condition, ARF activates the transcription factor p53 that triggers the expression of many apoptosis inducers and cell cycle inhibitory genes. *TP53* mutation is the most frequent genetic alteration in human cancers. In response to a wide variety of cellular stresses, p53 is able to induce the expression of target genes leading to cell cycle arrest, apoptosis or senescence. A transcriptional target of p53 is the MDM2 protein, which plays a central role in regulating p53 functions [35,36,37]. MDM2 is an E3 ubiquitin ligase that ubiquitinates p53 through its RING (Really Interesting New Gene) domain, common to many E3 ubiquitin ligases [38]. The two proteins are part of a negative feedback loop that keeps p53 levels low during normal growth and development. Interestingly, the dynamics of p53 activation appears to be the signal that dictates cell fate. While transient p53 activation induces cell cycle arrest, long-lasting p53 oscillations promote apoptosis [39]. The mechanisms that increase the stability and the activity of p53 point to alleviating the negative effect of MDM2 on p53. By interacting with p53, MDM2 blocks p53-mediated transactivation and targets the p53 protein for ubiquitin-dependent proteasome-mediated degradation [35,37,40,41]. Early studies reported that the p19ARF protein was involved in the p53 pathway via its interaction and inhibition of MDM2 [42,43]. Several studies then clarified that ARF inhibition of cell proliferation also relies on p53 independent pathways through the functional and molecular interaction with a number of different proteins (Figure 2).

### ARF Inhibits MDM2 Through Multiple Mechanisms

Several mechanisms have been formulated to explain ARF-mediated p53 stabilization and activation. Initial evidence reported that ARF is able to block the nucleus-cytoplasm shuttling of the MDM2-p53 complex [44]. ARF is predominantly located in the nucleolus in different cell lines and in different experimental conditions [45]. In regard to the human protein, this localization depends on two aminoacidic stretches, one located at the N-terminal region (aa 2–14), while the other resides at the C-terminal and comprises a sequence of basic amino acids (aa 82–101) [45,46]. The co-expression of ARF together with p53 and MDM2 blocks the export of both p53 and MDM2 to the cytoplasm. Furthermore, it was observed a re-localization of ARF from the nucleolus to the nucleus in sub-nuclear structures defined as nuclear bodies, in which p53 is stabilized and activated. Another mechanism reports that ARF induces p53 stabilization and activation by sequestering MDM2 in the nucleolus. This hypothesis is based on the evidence that the interaction between ARF and MDM2 reveals a cryptic nucleolar localization signal present in the C-terminal region of MDM2 [47]. This signal, in synergy with similar sequences within ARF, induces re-localization of the ARF-MDM2 complex in the nucleolus, thus preventing p53 binding and export to the cytoplasm [48,49].

An alternative mechanism, that probably integrates the previously described ones, reports that ARF, by MDM2 binding, inhibits its ubiquitin-ligase activity [50,51]. In order to be exported in the cytoplasm and degraded, p53 needs to be modified through the covalent binding to ubiquitin. Ubiquitination, in addition to targeting p53 to the proteasome for degradation, prevents the formation of p53 tetramers, thus inducing the exposure of the p53 nuclear export signals and its re-localization to the cytoplasm.

The observations that ARF mediated p53 stabilization can occur without relocation of MDM2 in the nucleolus, and that ARF mutants that do not exhibit nucleolar accumulation retain the ability to stabilize p53 [52], suggested that its nucleolar localization could contribute to, but is not essential for, p53 stabilization. As mouse and human homologues are poorly conserved it has been difficult to dissect which are the domains required for ARF localization, MDM2 binding and inhibition, and above all, how these aspects led to p53 stabilization. Moreover, the MDM2 binding and the p53 stabilization domains, as well as the nucleolar driving sequences, show a certain degree of overlap. In mice, for example, the domain mapped between aa 26–37 is not only required to induce p19ARF nucleolar localization but also for p53 accumulation and cell cycle arrest [53]. Another layer of complexity comes from the observation that N-terminally deleted p19ARF mutants, although impaired in p53 stabilization, are still able to induce its transcriptional activation and to inhibit cell growth. Conversely, expression of ARF mutants not able to block cell proliferation through p53 can still induce MDM2 accumulation in the nucleolus. Altogether these data point to the notion that MDM2 subcellular localization and p53 stabilization are not both required to guarantee p53-dependent ARF inhibition of cell growth, as previously supposed [54].

## 4. ARF is Involved In p53 Independent Pathways of Tumor Suppression

Although ARF is undoubtedly a critical component of the p53 pathway, there are many lines of evidence that ARF restrains cell growth independently of p53 (Figure 2). Analysis of mouse models in which *Arf*, *Trp53* and *Mdm2* genes were simultaneously ablated (triple knockout or TKO mice) showed a higher tendency towards tumor development than those lacking only p53 and MDM2 [55]. Furthermore, *Arf ^-/-^* and *Arf ^+/-^* mice develop a broader spectrum of tumors than *Trp53* null mice and ARF overexpression can induce cell cycle arrest in cells lacking p53. Finally, in cells derived from TKO mice, the reintroduction of ARF prevents S phase entry and/or trigger apoptosis by mechanisms that did not require the expression of p53 protein [55]. Similarly, in human lung tumor cells devoid of p53 expression, p14ARF induces cell cycle arrest accompanied by features of tumor regression [56]. Also, xenograft models of pancreatic cancer showed that ARF expression impedes cell colonization and thus metastasis formation in a p53-independent fashion [13]. The notion that ARF acts independently of the Mdm2–p53 axis in tumor surveillance is in line with the observation that ARF interacts with a multitude of different cellular partners, such as proteins involved in transcriptional control (E2Fs, DP1, p63, c-Myc, Hif1α), nucleolar proteins such as nucleophosmin (NPM/B23), viral proteins (HIV-1Tat), mitochondrial protein (p32) and others [10,57]. This variety of ARF’s interactors suggested that the protein could play a wide role in cell protection from different types of insults. In particular, for some targets, the interaction with ARF causes an alteration of their stability. For example, NPM/B23 and E2F become degraded by the proteasome in a ubiquitin-dependent manner upon ARF interaction. Other targets change their localization upon ARF expression, while others become activated or stabilized [58]. Moreover, it has been demonstrated that the interaction with the mitochondrial protein p32 determines ARF’s mitochondrial recruitment, through its domain in exon 2 (aa stretch 82–101) [59,60]. The ability to localize to the mitochondrion appears to be p53 independent and required for ARF-induced apoptosis. Interestingly, the ARF exon 2 encoded domain appears to have a role in the defense mechanism that protects cells from oxidative stress. In senescent melanocytes, endogenous ARF is constitutively expressed as a cytoplasmic protein recruited to dysfunctional mitochondria as part of a pathway aimed at maintaining low levels of intracellular superoxide [61]. ARF translocation to mitochondria induces dissipation of mitochondrial potential, with the consequent decrease of free radical levels and cell growth inhibition. This mitochondrial activity involves an evolutionarily conserved acidic motif GHDDGQ (residues 65–70 in p14ARF), which mediates the interaction with, and the consequent inhibition of, BCL-xL, a positive regulator of mitochondrial potential. This cell-protective mechanism appears to be targeted in familial melanoma thus explaining why ARF mutations predispose carriers to melanoma. This function appears to be p3-independent. Moreover, although the C-terminal domain is also required for ARF induced autophagy (as we will discuss below), it is also independent of ARF function in autophagy.

Another tumor-suppressive function of ARF relies on its ability to ensure chromosomal stability [1] through its functional interaction with Aurora B [62]. ARF ablation in primary mouse embryonic fibroblasts (MEFs) results in aneuploidy, caused by misaligned and lagging chromosomes and multipolar spindles. Albeit this appears to be a p53-independent ARF function, it has been shown that this mechanism can also follow p53-dependent routes [63].

It has been widely reported that ARF is able to promote the sumoylation of some of its interactors [64,65,66,67]. This modification modulates a variety of phenomena such as protein stability, transport, modulation of gene expression, ubiquitylation, DNA repair, and centromeric chromatid cohesion. ARF induces sumoylation of both MDM2 and NPM/B23 by direct interaction with these proteins and in a p53-independent manner [65,68,69]. In addition, it has been reported that ARF interacts with the Myc-associated zinc finger protein (Miz1) and, by inducing its sumoylation, facilitates the assembly of the Myc-Miz1 complex that assists the switch from G1 arrest to apoptosis [70]. It has been suggested that ARF promotes this process through direct interaction with the Small Ubiquitin Modifier (SUMO)-conjugating enzyme Ubc9 [65]. Other evidence of ARF involvement in sumoylation is derived from the observation that it can inhibit the function of a desumoylating protein, SENP3 [71]. Although ARF involvement in the sumoylation process is well documented, the biological meaning of ARF-mediated sumoylation is mostly still unclear. Upon thermal or oxidative stress, an increase of SUMO 2 and 3 levels has been reported. Cells over-expressing SUMO 2 and 3 exhibit premature senescence [72], and an increase of both p53 and pRb sumoylation [73,74,75]. Given the established role of ARF in senescence, it would be interesting to analyze ARFs role in sumoylation-induced senescence.

Intriguingly, while ARF mediates p53 stabilization, it has the opposite function with another member of the p53 family, the transcription factor p63 that plays a fundamental role in the development of epithelial derivatives [76,77]. ARF interacts with one of the p63 isoforms, namely DNp63α [78], and increase its sumoylation and its subsequent proteasome-mediated degradation in both tumoral and immortalized cell lines [79,80]. ΔNp63α has an established role in oncogenesis being expressed in several cancerous cells [81,82,83] and acting as dominant negative in respect to p53. ARF’s role in p63 degradation suggests that ARF while stabilizing p53 and eliciting p53-dependent pathways, at the same time can inhibit p63 oncogenic functions.

As a nucleolar protein ARF plays a role in both ribosomal biogenesis and control of cell mass growth [84,85]. It inhibits the formation of mature 28S and 18S ribosomal RNA [86], inactivates NPM, a key player of this pathway [67] and modulates both rRNA transcription [87] and processing [84,85].

## 5. Mechanisms Governing ARF Degradation

The study of ARF interacting partners has led to the accumulation of a bulk of knowledge about the mechanisms regulating its turnover within the cell. Both mouse and human ARF are relatively stable proteins in the nucleolus, with a half-life of about 6 h [88]. This suggested, and was lately experimentally demonstrated, that ARF nucleolar localization could be a mechanism to stabilize the protein [89]. Early studies provided evidence that ARF association with MDM2 in the nucleoplasm decreases its half-life [4,10,42] and that it rapidly decreases when the protein is forced to the nucleoplasm. In the nucleolus, nucleophosmin plays an important role in the control of ARF turnover [68,90]. In response to increased levels of NPM, the turnover of p19ARF is retarded, whereas NPM protein levels downregulation accelerates its degradation [88] and cause p19ARF exclusion from nucleoli. Both NPM binding and nucleolar localization are required to promote ARF stabilization [91,92,93,94]. Conversely, ARF causes NPM poly-ubiquitination and degradation [90,95,96].

For the vast majority of proteins, conjugation of ubiquitin to internal lysines is the initial event in their degradation by the ubiquitin-proteasome system (UPS). Although both human and mouse ARF proteins accumulate following treatment with proteasome inhibitors, early studies excluded the possibility that ARF turnover could be regulated by UPS because the human protein is lysine-less and the murine one has a single lysine residue not required for proteasome degradation [89,97,98]. Interestingly, it was observed that ARF is subjected to N-terminal ubiquitination, a rare mechanism of ubiquitin conjugation also displayed by MyoD (Myogenic Differentiation 1) [99] and p21 [100]. While ARF degradation is inhibited in cancer cell due to UPS dysfunction, in normal cell lines its half-life is strongly reduced [101]. Through biochemical purification, an ARF specific E3 ubiquitin ligase named TRIP12/ULF (Thyroid hormone Receptor Interactor Protein 12/Ubiquitin Ligase For ARF) promoting its N-terminal-ubiquitylation and degradation was identified. Oncogenic stress such as c-Myc and NPM overexpression both inhibit ULF-mediated ARF ubiquitylation [102].

ARF can also be degraded through ubiquitin-independent mechanisms. One of these mechanisms reports that the interaction with TBP-1 (Tat-Binding Protein-1) could cause ARF folding thus making this protein a poor substrate for proteasome destruction [98,103]. Another mechanism involves the 20S/REG-γ proteasome system [104] that resides in the nucleus of interphase cells and is involved in the degradation of small unstructured proteins, such as ARF, p21, and p16INK4a. While NPM prevents ARF degradation retaining it in the nucleolus, TBP-1 protects ARF mainly in the nuclear compartment.

## 6. ARF Plays a Role During Development and Differentiation

It has been observed that INK4-ARF expression increases with age, mirroring a decline in tissue regenerative capacity. In stem cells, the entire *INK4-ARF* locus is kept in an epigenetically silenced state due to the function of the Polycomb group of proteins [105,106]. It has been proposed that, as cells lose stemness and acquire differentiated features, the *INK4-ARF* locus is remodeled in order to become responsive to stress and mitogenic signals arising during cell differentiation [107,108]. Physiological expression of ARF during development has been described in a precise time frame during the differentiation of several tissue types, such as the developing eye, spermatogonia, during epithelial differentiation, and in the yolk sac. One of the first pieces of evidence for ARF involvement in development came from the observation that ARF knockout mice developed smaller eyes compared to wild type mice due to defects in the neuroretina and lens, resulting in blindness [109]. A closer examination revealed that the persistence of the platelet-derived growth factor (PDGF) signaling in *Arf ^-/-^* cells of the hyaloid vascular system (HVS) stimulates aberrant proliferation and survival of vitreal perivascular cells in the eye in a p53 independent manner. In line with this, *Pdgfr*β (Platelet-Derived Growth Factor Receptor β) deletion rescues the *Arf ^-/-^* eye phenotype and restores vision. Analysis of mouse models indicated that the role of ARF in hyaloid vascular regression is p53-independent [110,111]. Further studies showed that ARF induces the expression of several microRNAs (miR) that repress *Pdgfrβ* synthesis. In particular, the expression of miR-34 is required for both *Pdgfrβ* repression and cell proliferation arrest elicited by p19ARF [112].

Interestingly, Li and colleagues reported that ARF is physiologically expressed in the fetal yolk sac, a tissue derived from the extra-embryonic endoderm (ExEn) [113]. In in vitro cultured embryoid bodies, ARF expression regulates the late stages of ExEn differentiation through the induction of another microRNA, miR-205, that in turn induces a set of genes required for cell migration and adhesion. Interestingly miR-205 is also regulated by p53 (and by the other p53 family members, p63 and p73) [113].

A role played by ARF has been reported in male germ cell development in mice [111]. ARF is over-expressed in mitotically dividing spermatogonia lining the basement membrane of the seminiferous tubules. Undifferentiated spermatogonia evolve into spermatocytes during a spatio-temporal coordinated process by which cells detach from the basement of the tubules and through a series of mitotic and meiotic cellular divisions, move towards the lumen. In *Arf*-null progenitors, detachment triggers DNA damage and p53-dependent apoptosis (anoikis) thus resulting in a reduced number of mature sperm and testicular atrophy. These data surprisingly indicate that, in a physiological process such as differentiation of spermatocytes, ARF can actually prevent instead of inducing, p53-dependent apoptosis [114]. The exact mechanism(s) by which ARF contributes to germ cells genomic integrity during their maturation is still lacking. It has been reported that ARF levels increase upon treatment with ATM (ataxia-telangiectasia mutated) inhibitors, underlining its involvement in a defense pathway that protects cells upon DNA damage. Also, ARF involvement in chromosomal stability and its cross-talk with the machinery of homologous recombination during meiosis could be part of the mechanism underlining its role in differentiation. The continuing pursuit of ARF’s functions in development led to the discovery of cross-talk between the DNA damage response and angiogenesis [115,116,117]. An inverse correlation has been observed between the expression levels of the vascular endothelial growth factor (VEGF) and ARF leading to the hypothesis that ARF expression can inhibit angiogenesis both in physiological and pathological conditions. These aspects and the therapeutic approaches potentially deriving from these studies have been clearly reviewed in Kostinas et al., 2014 [116].

**Figure 3:**
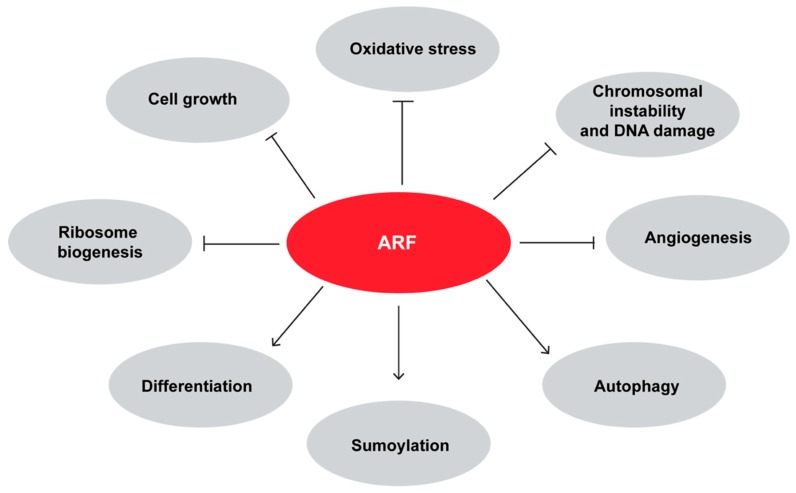
Synopsis of the effect of ARF in cellular processes. Note that the ARF negative effect on cell growth includes its ability to block cell proliferation and to induce apoptosis or senescence.

As previously mentioned, ARF interacts with and sumoylates p63 in keratinocytes. During differentiation, the proliferative cells of the basal level of epidermis detach, lose the ability to self-renew and start to express molecular markers of differentiating skin. In particular, p63 levels decrease whereas ARF protein levels increase at the onset of keratinocyte differentiation [79]. The increase of differentiation-dependent protein sumoylation machinery [118] led to a scenario in which ARF expression is required for p63 sumoylation and thus inactivation. Interestingly, it has been shown that p63 represses ARF promoter during development, and in line with this, ARF knockout rescues the severe epithelial phenotype of p63 null mice allowing both cell proliferation and their subsequent differentiation in keratinocytes [119]. Figure 3 shows a comprehensive picture of ARF involvement in several biological mechanisms.

## 7. ARF has Pro-Oncogenic Functions

ARF is found overexpressed in a significant fraction of human tumors, such as Burkitt’s lymphoma [120] and the majority of tumors with mutant p53 [43]. In 74 samples of aggressive B-cell lymphomas, p14ARF is mainly localized in the nucleoplasm and is associated with cancer progression and unfavorable prognosis. Also, at the transcriptional level, ARF expression is increased in patients with haematological malignancies, especially in the early stage of CML (chronic myelogenous leukemia), suggesting that this over-expression could be associated with CML development [121]. Inactivation of the *CDKN2a* locus is rare in thyroid follicular adenomas and carcinomas, where p14^ARF^ protein levels are strongly increased. Remarkably, ARF delocalization to the cytoplasm has been observed in thyroid aggressive papillary carcinomas, not accompanied by mutations in the ARF gene [122]. Although for several years ARF was considered to be dysfunctional when over-expressed in cancer tissues, surprisingly, ARF silencing was shown to limit the progression of Myc-driven lymphomas in mouse xenograft models [123]. In this study, Humbey and co-authors proposed for the first time that ARF can promote tumor progression through its ability to induce autophagy [60,124,125,126,127]. Autophagy is a lysosome-mediated process of self-digestion occurring during periods of nutrient deprivation. Target proteins and organelles are enveloped by the autophagosome, a double-membrane vesicle, that fuses with the lysosome leading to the degradation of its contents. The released amino acids are thus recycled for the synthesis of essential proteins [124]. ARF silencing in Myc-driven lymphoma cells impaired autophagy and decreased the ability of these cells to form tumors in vivo. In this cellular model, autophagy induction is thus a mechanism that helps cancer cells to survive during periods of metabolic or oxidative stress [123].

Interesting, a dual role in cancer has been proposed for autophagy [128]. The tumor suppressor function of this pathway has been inferred by the analysis of loss of function mouse models. Genetically engineered mouse models in which AuTophaGy essential genes (*ATG*s) were ablated show increased tissue and DNA damage, chronic inflammation, and chromosomal instability due to the accumulation of non-functional proteins and organelles and free radicals. This has been observed both in the liver and in the pancreas, tissues in which chronic inflammation creates a tumor-promoting environment [129,130,131]. Furthermore, the increased levels of the autophagosome cargo protein p62 upon dysfunctional autophagy promotes cancer survival by activation of the nuclear factor (erythroid-derived-2) like (NRF-2) a master regulator of the antioxidant defense response [132].

On the other hand, several observations showed that, in established tumors, autophagy functions as a survival pathway [133]. The metabolic survival function of autophagy is essential during starvation of all cells. However, some cancer cell lines have increased levels of autophagy also under non-starving conditions. In these cells, aberrant metabolic programmes are present in order to support hyper-proliferation and survival [134], cause the production of waste products, such as non-functional or misfolded proteins and DNA damage [135,136] that need to be discarded through the induction of autophagy. Moreover, activation of the antioxidant defense pathway induced by high levels of free radicals further promotes chemoresistance. During cancer evolution, the possibility to recycle cellular constituents is also an advantage for those cells that escape the primary tumor and disseminate through the body, a process that requires the ability to survive prolonged periods of starvation. In this scenario, it appears clear that while autophagy can contribute to ARF tumor suppression functions [137] the same mechanism can be hijacked to promote cancer evolution and survival [123].

Another evidence of an ARF pro-oncogenic role comes from the analysis of mouse models of prostate cancer. Genetic ablation of *p19Arf* in prostate epithelium does not accelerate but rather partially inhibits the prostate cancer phenotype of *Pten^-/-^* mice [138]. Tissue microarrays analysis performed on human prostate samples showed that p14ARF inactivation is indeed very rare in human prostate cancer and inversely correlated with disease aggressiveness. The authors observed that the double knock-out *Pten^-/-^/p19Arf ^-/-^* causes a decrease of prostatic intraepithelial neoplasia incidence in the ventral region of the prostate respect to *Pten^-/-^* mice. Interestingly, this effect appears highly cell type specific as mouse embryo fibroblasts (MEFs) derived from these mice show instead increased proliferation rate and present features of cellular transformation.

Using a similar approach, the analysis of *p19Arf* deletion was analyzed in the context of *Pten/Trp53* knockout mice [139]. In this study authors showed that ARF positively regulates prostate tumors growth by increasing the stability of the transcriptional factor Slug. Slug is a zinc-finger protein known to bind and repress the E-cadherin promoter thus mediating the so-called epithelial to mesenchymal transition (EMT), a cellular switch that provides cancer cells with the ability to disseminate through the body. In this study authors observed that in the nucleus of PC3 cells, ARF induces Slug stabilization by increasing its sumoylation. This results in the down-regulation of E-cadherin and, consequently, in the repression of the cell-cell junctions thus favoring cell migration and cancer metastasis. Both mathematical and experimental evidence suggested that the negative effect of Slug on E-cadherin can be counteracted by the expression of NRF2 [140]. In addition to a role in oxidative stress, it has been shown that NRF2 is required to allow collective cells migration, a hallmark of hybrid epithelial/mesenchymal phenotype (E/M). The hybrid E/M phenotype, a status characterized by the simultaneous presence of both epithelial and mesenchymal traits at morphological and molecular levels, has been suggested to be potentially more aggressive than a complete EMT, correlating with the acquisition of cancer stem-like traits, enhanced drug resistance and poor prognosis. Given the oscillation of NRF2 upon autophagy as previously discussed [132], and the regulative circuit between ARF and E-cadherin, it would be interesting to analyze how the interplay between these proteins functionally interact with the progression/establishment of the E/M phenotype. In a recent study, it was demonstrated that ARF is able to induce EMT in prostate cancer through the upregulation of metallopeptidase 7 (MMP-7), a secreted member of the peptidase family of matrix metalloproteinase (MMPs) [141,142]. The degradation of the extra cellular matrix (ECM) by matrix metalloproteinases favors cell detachment and promotes cell migration. Interestingly, the MMP-7 protein, shows ARF-dependent nuclear localization in *Pten/Trp53* deficient tumors, a feature of an aggressive cancer phenotype [143].

Recent findings in bladder cancer also unveiled a novel role of ARF in drug resistance. The majority of bladder cancers are no–muscle-invasive tumors with favorable survival outcomes. Although ARF expression is known to be elevated in bladder cancer, as a consequence of p53 loss-of-function, the *INK4a/ARF* locus has been found to be amplified and associated with poor survival in several cases of muscle-invasive bladder cancer (MIBC) [144]. An analysis of The Cancer Genome Atlas [145] showed that, in addition to *TP53* mutations, deletion or mutations of the *CDKN2A* locus has been found in 42% of cases [146]. Interestingly, a substantial percentage (35%) of MIBCs, displays mutant *TP53* without corresponding alterations of *INK4a/ARF* locus. In particular, ARF and not p16 appears significantly overexpressed in MIBC, and mainly localized in the nucleolus of patients-derived bladder cancerous cells [147], a feature that correlates with the tendency to develop chemoresistance. Using a mouse model in which the ARF coding region was conditionally ablated in the bladder tissue of *Trp53^-/-^/Pten^-/-^* mice, the authors showed that *Arf* wild-type tumors were markedly resistant to treatment with cisplatin compared with the *Arf*-null counterpart. Similarly, the overexpression of the human ARF protein in human bladder cancer cells and their use in orthotopic assays showed that it promotes cancer formation in vivo.

Additional support to ARF pro-proliferative role is provided by the observation that ARF interacts with the focal adhesion kinase (FAK) [67]. During cytoskeleton remodeling induced by cell spreading, ARF is recruited to cell periphery at points of focal adhesions, where it co-localizes and interacts with the active focal adhesion kinase (pFAKY^397^). Accordingly, ARF silencing induces cytoskeleton disorganization causing anoikis through a mechanism dependent on the death-associated protein kinase (DAPK). The DAPK is a pro-apoptotic serine/threonine kinase, activated following cytoskeleton–matrix disengagement [148]. The presented data suggest that p14ARF blocks DAPK functions avoiding DAPK-mediated cell death by both p53-dependent and -independent pathways [67].

Collectively this experimental evidence suggests that ARF has pro-survival functions, thus implying that it may function in both tumor suppressive and oncogenic pathways in cancers, depending on the genetic context.

## 8. ARF and Autophagy

Given the dual role of autophagy in cancer, the involvement of ARF in autophagy could be the key to interpret ARF role in cancer as well. Early evidence of ARF role in autophagy led to the identification of a shorter isoform of the protein translated by an internal methionine residue (Met 45 in mice and Met 48 in humans), which is not conserved in other ARF orthologs. Translation from this residue produces the so-called smARF (short mitochondrial ARF), which lacks the well-conserved N-terminus end, displaying most of the known p53-dependent functions of the protein. In response to viral and cellular oncogenes, smARF is upregulated and localizes to mitochondria, where it is stabilized by the interaction with the mitochondrial protein p32 [149]. Here, smARF induces the dissipation of mitochondrial membrane potential not accompanied by cytochrome c release to the cytoplasm, suggesting that there is no apoptotic cell death. In line with this, overexpression of smARF in 293T cells causes caspase-independent cell death characterized by the accumulation of autophagic vacuoles. Interestingly, the knockdown of autophagy essential genes such as *Atg5* or *Beclin-1* attenuates cell death in smARF-expressing cells, leading to the hypothesis that autophagy contributed to the observed process [60,150]. However, the smARF localization to mitochondria has been related to its ability to induce a selective form of autophagy called mitophagy, a cellular process that specifically removes damaged or excessive mitochondria [125,137,151,152]. Although mitophagy shares many features with autophagy, the selectivity of the autophagosome for mitochondrial cargo is due to a set of proteins that are activated under specific stimuli. Indeed, smARF recruitment to mitochondria induces membrane depolarization that in turn, provokes the stabilization of the mitochondrial PINK1 kinase and the PINK1-mediated recruitment of the Parkin ubiquitin ligase on the outer membrane of the mitochondrion. Parkin, by ubiquitinating several mitochondrial proteins on the outer membrane, primes the mitochondrion for subsequent degradation through the autophagosome [151]. Further complexity has been added following the observation that smARF can have a negative role on stress-induced mitophagy, a process mediated by the JNK2 kinase [153]. Authors observed that JNK2 induces the ubiquitination and the proteasome-mediated degradation of smARF upon stress. In this condition, basal autophagy (smARF dependent) is impaired and the protein levels of p62 increase. This protein, recruited to the mitochondrial membrane, mediates the formation of the autophagosomal vesicles thus promoting the selective degradation of mitochondria. Interestingly, smARF re-expression in ARF null mice rescues the ocular defects and the reduction of sperm production, thus suggesting that these developmental functions depend on smARF dependent functions [149].

It has been now also extensively shown that full-length ARF proteins (both human and mouse) can promote autophagy in a p53-dependent and independent manner [137]. Mitochondrial localized full-length ARF physically interacts with BCL-xL, a member of the Bcl2 family, that plays an important role in autophagy. The autophagy-inducing activity of ARF was shown to involve the displacement of Beclin-1 from BCL-xL complexes through direct binding of ARF to BCL-xL [124,154,155,156,157,158,159,160,161,162] thus promoting the activation of the key mediator of autophagy, the Beclin-1/Vps34 complex [124,154,155].

Analysis of ARF mutants showed that the ARF C-terminal domain, comprising amino acids 100 to 120, is necessary for autophagy induction by both human and murine protein [125]. Natural melanoma mutations in exon 2 (R98L, L104I and R115G), affecting the coding potential of ARF, but not of p16INK4a, impair ARF ability to induce autophagy [125]. Interestingly, a domain coded by exon 2, within the 100–132 aminoacidic stretch, has been shown to promote both FAK activation and cell survival thus suggesting the speculation that ARF-mediated autophagy could be, at least in part, the mechanism underlining its role in cell spreading [67]. In support of this hypothesis, in cells that detach from the substrate, or in cells with low level of activated FAK, autophagy inhibition induces anoikis [156]. Indeed autophagy, by protecting matrix-detached epithelial cells from anoikis could promote metastatic processes [157]. In support of the cross-talk between cell morphology and autophagy-mediated survival, ATG proteins have been shown to co-localize with FAK at focal adhesions [156]. Moreover, autophagy regulates cell spreading, especially cell protrusions extensions [158], and is required for focal adhesion turnover and cell migration [159] further supporting the view that ARF could promote cell spreading through its ability to induce autophagy. It should be underlined, that while the expression of the N-terminal ARF domain is impaired in mediating cell spreading, the C-terminal region, in which the autophagy-promoting domain has been mapped, completely rescues the morphology defect of ARF silenced HeLa cells [67]. Further experiments will be necessary to validate this hypothesis and clarify the possible involvement of autophagy in this ARF function and/or in tumor progression. A summary of ARF dependent functions is provided in Table 1.

## 9. Conclusions

Mammalian cells sustaining oncogenic insults invoke defensive programs resulting either in cell growth arrest or apoptosis. In non-pathological conditions, ARF protein level is almost undetectable within the cell. Upon oncogenic stimuli or developmental cues, its levels increase, eliciting p53-dependent or independent pathways to restrain cell proliferation. ARF protein interacts with a cohort of different partners involved in different cellular processes.

Recent experimental evidence, also pursued by the observation of high ARF protein levels in specific tumors, suggested that this protein might have pro-survival functions. The possibility that ARF might promote or improve the survival of a subset of tumors has been at least in part correlated with ARFs ability to induce autophagy. Experimental data suggest that within a tumor cell, ARF protein can be stabilized by decreased proteasome-mediated degradation [101] or by protein kinase C (PKC) activation [160]. In this latter case, ARF phosphorylation mediates its accumulation in the cytoplasm where it cannot efficiently exert its classical role in the control cell proliferation [160,161]. Although this can be a way to escape ARF surveillance in tumorigenesis, it might likewise mean that phosphorylation can confer pro-survival properties to the protein favoring cancer progression. In line with this last hypothesis, the over-expression of an ARF mutant mimicking the phosphorylated status of the protein promotes proliferation in HeLa cells, induces FAK stabilization and allows cell spreading [162]. Adhesion/spreading interplay has a well-documented role not only in malignancy but also in physiological processes such as embryogenesis [113]. Interestingly ARF involvement in cytoskeleton organization relies on an evolutionarily conserved mechanism, as p19ARF is able to rescue spread morphology defects caused by p14ARF silencing. The ARF exon 2-encoded region (aa 65–132) which, compared to the exon 1β shows a stronger degree of identity between human and its mouse homologue, is required for FAK binding, activation and cell spreading. These observations add a crucial element in the understanding of the functions of the exon 2 encoded domain, in which the bulk of ARF mutations in human cancers resides. The data presented thus implies that the cellular environment, both within and outside the cell, can dictate the function of ARF as a tumor suppressor or as a pro-metastatic player in cancer. Unravelling the molecular mechanisms underlying ARF duality in cancer progression will be of great interest for the future design of cancer therapy.

## Figures and Tables

**Table 1:** ARF roles in cell proliferation and molecular mechanisms of the observed function.

Functions	Mechanisms
**Tumor Suppression**	
Growth control	p53 dependent and independent call cycle arrest and apoptosis. ΔNp63 inhibition [4,5,11,12,13,31,32,33,34,42,43,55,79,80]
Chromosomal stability	Stabilization of miotic spindle, prevents aneuploidy [62]
Ribosomal biogenesis	Inhibition or rRNA processing and transcription [84,85,86,87]
Oxidative stress	Cellular protection from dysfunctional mitochondria [61]
DNA damage	Activation of ATM/ATR/CHK pathway, p53-dependent pathways of DNA repair [115,116]
**Contest Dependent**	
Autophagy	Beclin-1 activation, dissipation of mitochondrion potential [60,123,124,125,126,127]
Differentiation	Inhibits angiogenesis in developing eye, protects from apoptosis in spermatogonia, allows extraembryonic endoderm migration [79,110,111,112,113,114,115,116,117]
**Tumor Promoter**	
Epithelial to mesechimal transition	Slug stabilization [139]
Modulation of tumor microenvironment	Metallopeptidase-1 interaction [141]
Survival of lymphoma, protease and bladder tumor cells	Promotes prostate cancer in Pten mouse model and autophagy in lymphomas; promotes chemoresistance in bladder cancer [120,121,122,123,124,125,126,127,138,141,147]
Anoikis protection, increased proliferation	Inhibition of DAPK mediated cell death; activation focal adhesion kinase [67,160,161,162]
